# A Transcriptome Approach Evaluating the Effects of Atractylenolide I on the Secretion of Estradiol and Progesterone in Feline Ovarian Granulosa Cells

**DOI:** 10.3390/vetsci11120663

**Published:** 2024-12-17

**Authors:** Yuli Guo, Junping Liu, Shuangyi Zhang, Di Sun, Zhiying Dong, Jinshan Cao

**Affiliations:** Key Laboratory of Clinical Diagnosis and Treatment Techniques for Animal Disease, College of Veterinary Medicine, Inner Mongolia Agricultural University, Hohhot 010018, China; yuliguo@emails.imau.edu.cn (Y.G.);

**Keywords:** ovarian granulosa cell, *Atractylenolide* *I*, estradiol, progesterone, cholesterol metabolism

## Abstract

*Atractylodes macrocephala Koidz* (AMK) as an oriental medicine has been used in the treatment of threatened abortion. *Atractylenolide I* (AT-I) is one of the major bioactive components of AMK. This study aimed to investigate the effect of AT-I on the secretion of estradiol (E_2_) and progesterone (P_4_) in feline ovarian granulosa cells (FOGCs), which is necessary for pregnancy. At first, the proliferation of FOGCs after AT-I treatment was measured by CCK-8. Then, the synthesis of E_2_ and P_4_ were measured by ELISA. Lastly, transcriptome sequencing was used to detect the DEGs in the FOGCs, and RNA-seq results were verified by RT-qPCR and biochemical verification. It was found that AT-I could promote proliferation and the secretion of E_2_ and P_4_ in FOGCs; after AT-I treatment, 137 significant DEGs were observed, out of which 49 were up-regulated and 88 down-regulated. The DEGs revealed significant enrichment of 52 GO terms throughout the differentiation process (*p* < 0.05), as deciphered by Gene Ontology enrichment analysis. Kyoto Encyclopedia of Genes and Genomes analysis manifested that the DEGs were successfully annotated as members of 155 pathways, with 23 significantly enriched (*p* < 0.05). A relatively high number of genes were enriched for the cholesterol metabolism, ovarian steroidogenesis, and biosynthesis of unsaturated fatty acids. Furthermore, the contents of the total cholesterol and low-density lipoprotein cholesterol were decreased by AT-I treatment in the cell culture supernatant. The results indicated that AT-I could increase the ability of FOGCs to secrete E_2_ and P_4_, which might be achieved by activation of cholesterol metabolism.

## Simple Summary

Atractylodes macrocephala Koidz (AMK) as an oriental medicine has been used in the treatment of threatened abortion. Atractylenolide I (AT-I) is one of the major bioactive components of AMK. This study aimed to investigate the effect of AT-I on the secretion of estradiol and progesterone in feline ovarian granulosa cells (FOGCs), which is necessary for pregnancy. It was found that AT-I could promote proliferation and the secretion of estradiol (E_2_) and progesterone (P_4_) in FOGCs; after AT-I treatment, 137 significant DEGs were observed, out of which 49 were up-regulated and 88 down-regulated. A relatively high number of genes were enriched for the cholesterol metabolism, ovarian steroidogenesis, and biosynthesis of unsaturated fatty acids. Furthermore, the contents of the total cholesterol and low-density lipoprotein cholesterol were decreased by AT-I treatment in the cell culture supernatant. The results indicated that AT-I could increase the ability of FOGCs to secrete E_2_ and P_4_, which might be achieved by activation of cholesterol metabolism.

## 1. Introduction

Feline reproduction has not received a great amount of investigation and attention [1]. Abortion is among the primary reasons for pregnancy termination in felines. Within this area of research, hormonal and antibiotic therapies are predominantly utilized [2,3]. However, there has been a surge in reports detailing embryonic malformations, infertility, and various side effects resulting from the overuse of hormones and antibiotics. Thus, the creation of secure and potent fetal safeguarding products is crucial for their clinical utility in diminishing the rate of feline abortions and for the conservation of elite breeds.

*Atractylodes macrocephala Koidz* (AMK) has been commonly used in the clinical prescription for preserving fetuses, and its fetal safety effects have been confirmed by thousands of years of medical practice in traditional Chinese medicine [4]. As one of the major bioactive components in AMK, *Atractylenolide I* (AT-I) is a naturally occurring sesquiterpene lactone that has been found to display a wide range of pharmacological and biological activities such as anti-atherosclerotic activity and the treatment of various inflammatory illnesses [5,6,7,8].

Progesterone (P4) has a variety of physiological functions, such as promoting endometrial decidualization, establishing maternal–fetal immune tolerance, inhibiting inflammatory responses, and improving uterine–placental circulation, which are prerequisites for the maintenance of pregnancy [9,10]. Insufficient secretion of P_4_ affects endometrial secretion, thereby affecting embryo implantation and development. In early embryonic development, P_4_ mainly comes from luteal granulosa cells, so the activity of granulosa cells plays a very important role in corpus luteum (CL) development and early pregnancy maintenance [11,12]. The CL is the primary provider of progesterone in felines, yet the placenta of cats may contribute to the maintenance of pregnancy through local paracrine or autocrine processes [13]. Maintaining a certain concentration of estradiol (E_2_) can increase the uterine placental blood flow and increase the sensitivity of the uterus to P_4_ [14]. E_2_ plays a role in the endometrium mainly through its homologous nuclear receptors ESR1 and ESR2, and participates in the decidualization process of the endometrium through the ERK/MAPK pathway [14]. Studies have shown that if the E_2_ content is too low, it would lead to embryo implantation failure [15]. Therefore, determining the effect of AT-I on the secretion of E_2_ and P_4_ in feline ovarian granulosa cells (FOGCs) might contribute to our understanding of the protection of AMK in early pregnancy.

In recent years, the high-throughput RNA sequencing technique (RNA-seq) has emerged as a powerful tool for transcriptome analysis [16]. By using this method, it might help to assess the changes in the transcriptome in FOGCs upon treatment with AT-I at the whole genome scale, contributing to explaining the mechanism of how AT-I regulates the changes in FOGCs.

## 2. Materials and Methods

### 2.1. AT-I Structure

**Figure 1:**
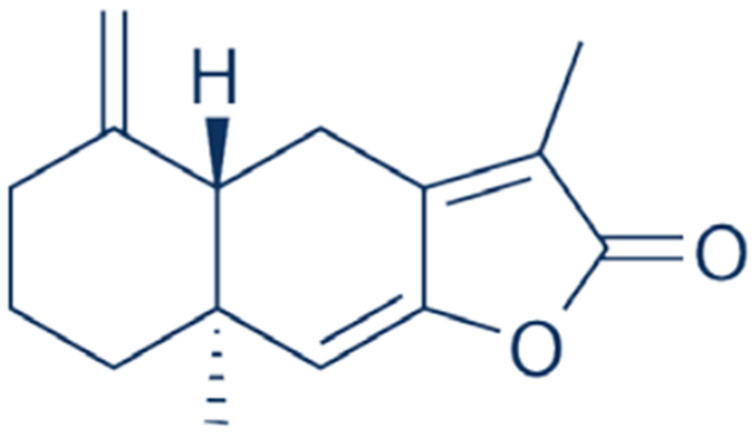
Chemical structure of Atractylenolide-I (AT-I).

The AT-I (CAS No. 73069-13-with purity ≥ 98%) used in this study was purchased from Dalian Meilune Biotechnology Co., Ltd. (Dalian, China). The chemical structure of the AT-I is depicted in Figure 1.

### 2.2. Culture of Primary Feline Ovarian Granulosa Cells

FOGCs were obtained from fresh ovarian tissues of healthy and diestrus period felines between six months and two years old, which were under sterilization operation in the animal hospital of Inner Mongolia Agricultural University. The feline ovarian tissues were maintained in a vacuum thermos flask containing sterile physiological saline and transported to the laboratory within 2 h. The ovarian tissues were washed several times with 37 °C DPBS solution (Gibco, San Diego, CA, USA), which contained 3% penicillin and streptomycin (Gibco, San Diego, CA, USA), until the tissues were completely clean. The tissues were then transferred to a 300 mm diameter plate with 0.5 mL of 12% fetal calf serum-supplemented Ham’s F-12 nutrient mixture (DMEM/F12, Gibco, Grant Island, NY, USA). Thereafter, the follicular fluid and cumulus–oocyte complexes (COCs) from primary follicles between 1 and 1.5 mm in diameter were aspirated by using a 2.5 mL aseptic syringe with a needle size of 0.45 × 13 mm. Collected cells were transferred to a 1 mL centrifuge tube and centrifuged at 503.1× *g* for 3 min at 23–25 °C; then, the sediment was harvested and washed with DPBS solution. This process was repeated three times. Afterward, the isolated FOGCs were transferred to a 25 cm^2^ cell culture bottle (Thermo, Waltham, MA, USA), and cultivated in a 5% CO_2_ incubator at 37 °C. Cell growth and morphological characteristics were observed with an inverted microscope (Philips, Tokyo, Japan). After 72 h, the oocytes were washed out with DPBS at 37 °C, and the granulosa cells were isolated. Then, the medium was renewed routinely every other day until the cell density reached 75–80% (normally 3–4 days). Then, it was digested with 0.25% trypsin-EDTA (Gibco, San Diego, MA, USA) of 1 mL for 2 min, and the digestion was terminated with the DMEM/F12 culture medium containing 12% serum. After fully mixing, it was inoculated at a split ratio of 1:2 for subsequent cultivation.

### 2.3. Morphological Observations

The FOGCs at the logarithmic growth phase were seeded 100 µL at a density of 1 × 10^5^ cells/mL on the autoclaved slides. Thereafter, the slides were stored in an autoclaved wet box that was placed in a 37 °C, 5% CO_2_ incubator for 24 h. After reaching 70% confluence, the slides were washed three times with DPBS and the cells were fixed for 20 min with cold acetone. The processed slides were then stained with two different staining methods: hematoxylin-eosin (HE) and Wright–Giemsa. They were then photographed and analyzed by using a light microscope (ECLIPSE TS100-F. Nikon, Tokyo, Japan).

### 2.4. Immunofluorescence Identification

To analyze the identity and purity of isolated FOGCs, immunofluorescence staining for a specific marker of ovarian granulosa cells was performed by FSHR (follicle-stimulating hormone receptor). An amount of 0.5 mL of the logarithmic growth phase FOGCs were seeded at a density of 1 × 10^5^ cells/mL into 35 mm glass-bottom dishes (Shengyou Biotechnology, Hangzhou, China). After the cells reached 80% confluence, they were washed three times with aseptic DPBS solution, fixed for 30 min with 4% paraformaldehyde (Solarbio, Beijing, China) at 23–25 °C, and washed three times with DPBS. After fixation, the FOGCs were incubated with 1 mL 0.25% Triton X-100 (PH = 7.40, Sigma-Aldrich, St.Louis, MO, USA) for 20 min, and then washed three times with DPBS. After blocking for non-specific binding sites by BSA, the cells were stained with a primary antibody to the FSHR (1:200, Rabbit Anti-FSH receptor antibody; BS-0895R; Bioss; Beijing, China) overnight at 4 °C. The granulosa cells were washed three times in DPBS and incubated with a secondary antibody (Alx647; donkey-anti-rabbit IgG; Jackson, PA, USA) for 2 h at 23–25 °C in the dark. After incubation, the cells were washed three times for 10 min with DPBS in the dark. Then, the nuclei were stained with DAPI, and washed on the decolorizing shaker four times for 10 min. Glass-bottom dishes were observed under an LSM 800 (Zeiss, Jena, Germany) laser confocal system.

### 2.5. Cell Viability Assay

The cell viability was assessed using Cell Counting Kit-8 assays (CCK-8; BS350B; Biosharp; Shanghai, China). The second-generation feline ovary granule cells were used, washed with DPBS, and 1 mL of 0.25% trypsin–EDTA was added to digest them for 2 min. Thereafter, the DMEM/F12 medium containing 12% fetal bovine serum (Excell Biology, Shanghai, China) was added to stop the digestion. The pipetting was repeated to ensure that the cells are away from the wall and uniformly distributed. After digestion, the cell suspension concentration was adjusted to 2 × 10^6^ cells/mL. Thereafter, a 96-well cell culture plate (Shengyou Biotechnology, Hangzhou, China) was taken, 100 µL of culture medium with cells was added to each well, and the plate was incubated for 12 h. Thereafter, 100 µL DPBS was added to the remaining wells to reduce evaporation. The cells in the plate were purified for 6 h (with the serum-free medium), the cell supernatant was purged after purification, and 100 µL medium (containing 12% serum) was added. The plate was cultivated for 6 h, 12 h, 24 h, 36 h, and 48 h, and then CCK-8 was added for 4 h. After then, the absorbance value (OD value) of each well was measured with a microplate reader at a wavelength of 450 nm.

### 2.6. Cytotoxicity of AT-I Against Primary Feline Ovarian Granulosa Cells

To obtain a well-cultured cell culture dish as described in Section 2.5, after the FOGCs were purified as described above, 100 µL medium (containing 12% serum) with different AT-I concentrations (0 µmol/L, 1 µmol/L, 3 µmol/L, 10 µmol/L, 30 µmol/L, 100 µmol/L, and 300 µmol/L) was added to the 96-well cell culture plate (Shengyou Biotechnology, Hangzhou, China) and incubated at 37 °C for 0 h, 12 h, 24 h, 36 h, and 48 h. CCK-8 was added for 4 h and the absorbance value of each well was measured with a microplate reader at a wavelength of 450 nm to measure the cytotoxicity.

### 2.7. Determination of Estradiol (E_2_) and Progesterone (P_4_) Concentration

The second-generation FOGCs were seeded at a density of 2 × 10^6^ cells/mL in 6-well cell culture plates (Shengyou Biotechnology, Hangzhou, China) in the DMEM/F12 medium containing 12% fetal bovine serum (Excell Biology, Shanghai, China) and 1% streptomycin and penicillin and cultivated at 5% CO_2_ at 37 °C until 70% of the cells were fused. The cell culture supernatant was then removed and 2 mL of fresh DMEM/F12 medium containing 10 µmol/L AT-I was added to the FOGC monolayer for co-culturing at 37 °C with 5% CO_2_. After incubation for 0 h, 12 h, 24 h, 36 h, and 48 h, respectively, the supernatant was aspirated and used for subsequent experiments. The control cells were treated with the DMEM/F12 medium with the same time condition. Three distinct biological replicates were set up for both the treatment and control experiments.

The amount of E_2_ within the culture medium was evaluated by means of an enzyme-linked immunosorbent assay (Wuhan Xinqidi Biological Technology; Wuhan, China) according to the manufacturer’s instructions. The estradiol standard curve ranged from 0 to 1000 pg/mL. The samples were analyzed in triplicates. The assay sensitivity, range, and intra-assay coefficient of variation were 5 pg/mL, 15.6–1000 pg/mL, and ≤8%, respectively. The concentrations of P_4_ in the culture medium were measured by an enzyme-linked immunosorbent assay (Wuhan Xinqidi Biological Technology; Wuhan, China) according to the manufacturer’s instructions. The progesterone standard curve ranged from 0 to 100 ng/mL. The samples were analyzed in triplicates. The assay sensitivity, range, and intra-assay coefficient of variation were 0.5 ng/mL, 1.56–100 ng/mL, and ≤8%, respectively.

### 2.8. AT-I Treatment

According to the above experiments, we selected the DMEM/F12 medium containing 12% fetal bovine serum (Excell Biology, Shanghai, China) with 10 µmol/L AT-I, and incubated it for 36 h for the next study. FOGCs were seeded at a density of 2 × 10^6^ cells/mL in 6-well cell culture plates and cultivated at 37 °C and 5% CO_2_ until they reached 70% confluence. The supernatant from the cell culture was then taken off, and the FOGC monolayer received a fresh 2 mL addition of DMEM/F12 medium with 10 µmol/L AT-I. The plate was subsequently placed in an incubator for 36 h, maintaining a temperature of 37 °C and a CO_2_ concentration of 5%. After AT-I treatment, FOGCs were photographed and analyzed for potential morphological changes using an inverted microscope (Philips, Tokyo, Japan). The supernatant was aspirated and used for subsequent experiments. Subsequently, the FOGCs underwent a meticulous triple wash with DPBS to guarantee the complete removal of any residual drug and the cells were then prepared for transcription analysis. The control cells received treatment exclusively with the DMEM/F12 medium. Both the treated and control cells were assessed using three biological replicates to validate the consistency of the experimental outcomes.

### 2.9. RNA Library Construction and Sequencing

The total RNA from untreated and AT-I-treated FOGCs was extracted according to the instructions of the Axygen-RNA extraction kit (AxyGen, Hangzhou, China), and there were three biological repeats in each group. Bioanalyzer 2100 and RNA 6000 Nano LabChip Kit (Agilent, Santa Clara, CA, USA) were thereafter used to analyze the quantity and purity of total RNA, yielding RIN scores > 8.0, in accordance with the testing standard. Magnetic beads connected with Oligo were used to enrich and purify eukaryotic mRNA with a poly-A tail. The extracted eukaryotic mRNA was randomly broken into short fragments by the fragmentation reagent (Fragmentation Buffer). Using the fragmented mRNA as the template, one-strand cDNA was synthesized by a six-base random primer (Random hexamers), and then the buffer, dNTPs, RNaseH, and DNA Polymerase I were added to synthesize the two-strand cDNA. The cDNA double-stranded product was purified by AMPureXP beads. The viscous end of the DNA was repaired to a flat end by T4 DNA polymerase and Klenow DNA polymerase, and the 3’ end was selected by adding base An and spliced AMPureXP beads. Finally, the final sequencing library was obtained by PCR amplification. After passing the quality inspection of the library, the library was sequenced with Illumina Hiseq 4000 to produce 150 bp double-terminal data. The cDNA library was sequenced on an Illumina HiSeq 4000 sequencing platform (Illumina, San Diego, CA, USA) provided by Majorbio Bio-pharm Technology Co., Ltd. (Shanghai, China).

### 2.10. Statistical Analysis of Transcription Data

The raw RNA-seq data were then filtered to remove various adaptor contamination, low-quality bases, and undetermined bases utilizing Cutadapt software (Version 3.3) [17]. The sequencing quality was authenticated through the utilization of FASTQC software (Version 0.11.6), which assessed the Q20, Q30, and GC content of the clean data. All analyses that followed were performed on the basis of these clean, high-quality data. The filtered reads were then aligned and mapped to the reference genome using Hisat 2.0. The aligned read files were processed using Cufflinks [18], which uses the normalized RNA-seq fragment counts to measure the relative abundances of the different transcripts. The unit of measurement is fragments per kilobases of exon per million fragments mapped (FPKM). The DEGs were screened using R package edger [19] with a fold change (FC) ≥ 1.5 and *p-adjust* < 0.05, which were considered significantly different expressed [20]. The DEGs underwent enrichment analysis using GO and KEGG. *p* values were calculated using the Benjamini-corrected modified Fisher’s exact test, and *p* < 0.05 was deemed statistically significant.

### 2.11. Validation by Quantitative Reverse Transcription PCR

Quantitative reverse transcription PCR (RT-qPCR) was utilized to validate the DEGs identified by RNA-seq. The total RNA concentration of all samples was adjusted to 300 ng/μL at the same time, and cDNA was used as starting material for a real-time PCR with FastStart Universal SYBR Green Master (Roche, Mannheim, Germany) on an iQ5 multicolor real-time PCR detection system (Bio-Rad, Houston, TX, USA). Eight DEGs were chosen based on changes in their expression levels in the treated cells as compared with the control cells. Eight DEGs were selected with cDNA as a template and the glyceraldehyde 3-phosphate dehydrogenase (*GAPDH*) gene was used as the internal control. The sequence of primers is shown in Table 1. The RT-qPCR reaction conditions are as follows: 5 min pre-incubation at 95 °C; 40 cycles of amplification for 5 s at 95 °C for denaturation; 34 s at 60 °C for annealing; and 20 s at 72 °C for elongation. Negative controls (without cDNA) were run in the same reaction set. The relative mRNA expression of DEGs in each group was calculated by the 2^−ΔΔCt^ formula [21].

### 2.12. Biochemical Verification

After 10 µmol/L AT-I treatment for 36 h, the supernatant was collected for biochemical testing (IDEXX ProCyte Dx; Westbrook, ME, USA). Three biological replicates were used for the treatment and control experiments. The contents of total cholesterol (TC) and low-density lipoprotein cholesterol (LDL-C) in the supernatant were detected to explore the possible effect of AT-I on lipid metabolism.

### 2.13. Statistical Analysis

The experimental data were expressed as mean ± SD. The difference between the two groups was analyzed using the *t*-test and One-way ANOVA. *p* < 0.05 was considered significant with *, *p* < 0.05 and **, *p* < 0.01.

## 3. Results

### 3.1. Identification of Cultured Feline Ovarian Granulosa Cells

**Figure 2:**
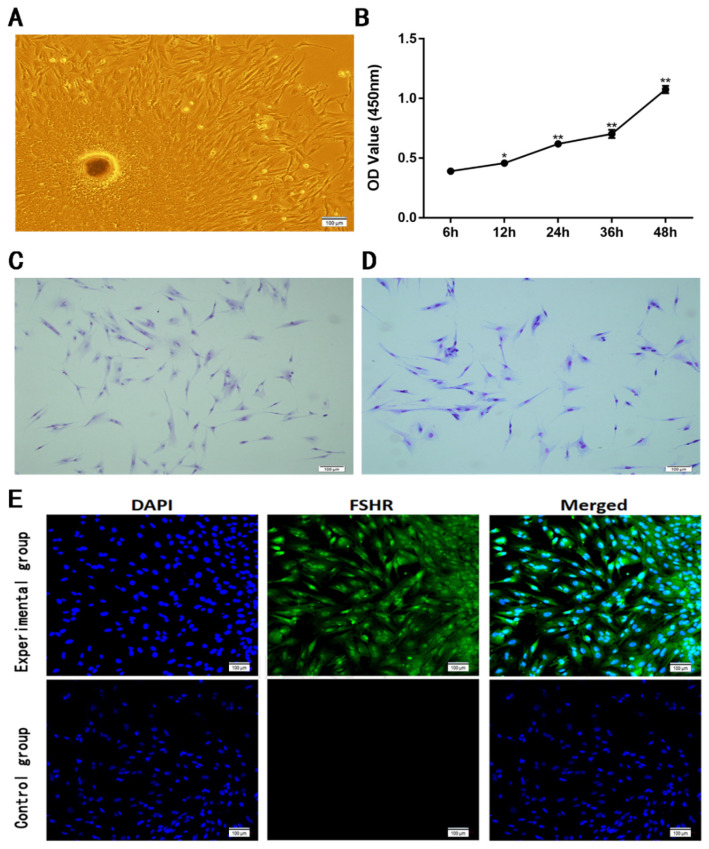
Morphological observation and growth curve of the primary FOGCs. (**A**) The primary FOGCs attach to the substrate around the oocytes and begin to display an elongated or fibroblastic property within the first 24 h of culture. (**B**) Growth curve of cultivated cells within 48 h. (**C**) The HE staining of cultivated cells on the substrate. (**D**) The Giemsa staining of cultivated cells on the substrate. (**E**) Immunofluorescence of cultivated cells on the substrate: FSHR (green) as the marker expressed in FOGCs; negative-control group and nuclei with DAPI staining (blue). Values are expressed as mean ± S.D. (n = 4). *, *p* < 0.05; **, *p* < 0.01.

The cells near the oocytes began to display an elongated or fibroblastic property within the first 36 h (Figure 2A). The cells were then distinguished by the presence of a triangle cone or irregular star-shaped morphology. After 3 days of culture, a large number of cells were observed around the oocytes in clusters. On the seventh day, the cells’ confluence reached between 75% and 80%. After sub-culturing, the cells started adhering within four hours, and by the third day they had achieved a confluence of 80%. The similar phenomenon happened on passages 3 and 4 with a classic irregular star-shaped morphology.

By HE staining, the structure of cells was confirmed to be contact by a clear edge and a triangle cone or irregular star shape. The cytoplasm was pink, and the nuclei were dyed in blue (Figure 2C). By Giemsa staining, the structure of cells was also presented as contact by a clear edge and a triangle cone or irregular star shape. The cytoplasm was blue-violet and the nuclei were pink (Figure 2D).

The FSHR was expressed in the cytoplasm of triangle cone-like cells by immunofluorescence staining, while the FSHR was not observed in the control group (without a primary antibody) (Figure 2E), confirming that FOGCs were cultivated successfully and the purity could reach 90%.

### 3.2. Cell Viability Test Results

The cell growth curve detected by the CCK-8 assay (Figure 2B) showed that cell proliferation significantly happened after 12 h until 48 h (*p* < 0.05), indicating that cultivated FGOC growth is consistent with normal cell growth.

### 3.3. Cytotoxicity of AT-I on the Primary FOGCs

**Figure 3:**
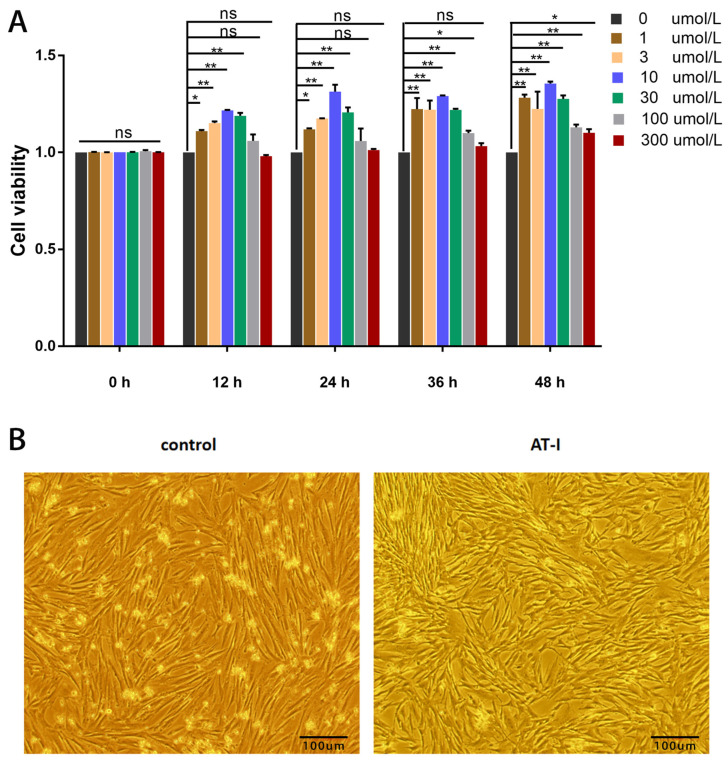
Effect of AT-I on the FOGCs. (**A**) Treatment with AT-I in 1, 3, 10, and 30 µmol/L could effectively promote FOGC proliferation from 12 h to 48 h; 100 µmol/L significantly increased FOGC proliferation after 36 h and the increased effect of 300 µmol/L happened at 48 h (*p* < 0.05). (**B**) After 10 μmol/L was AT-I-treated for 36 h, the density of granulosa cells increased, and cell viability increased significantly. Values are expressed as mean ± S.D. (n = 4). *, *p* < 0.05; **, *p* < 0.01; ns, not statistically significant.

AT-I treatment in a concentration range of 0 µmol/L–300 µmol/L showed that 1, 3, 10, and 30 µmol/L could effectively promote FOGC proliferation from 12 h to 48 h (Figure 3A). An amount of 100 µmol/L significantly increased FOGC proliferation after 36 h and the increased effect of 300 µmol/L happened at 48 h (*p* < 0.05) (Figure 3A). In addition, under the microscope, the increased density of granulosa cells by 10 µmol/L AT-I treatment for 36 h was clearly observed as well (Figure 3B). These results indicated that 10 µmol/L AT-I could enhance the viability at 36 h most obviously among selected concentration and time points, indicating that 10 µmol/L could be used as the experimental concentration for the following experiment.

### 3.4. The Effect of AT-I on the Synthesis of E_2_ and P_4_ in FOGCs

**Figure 4:**
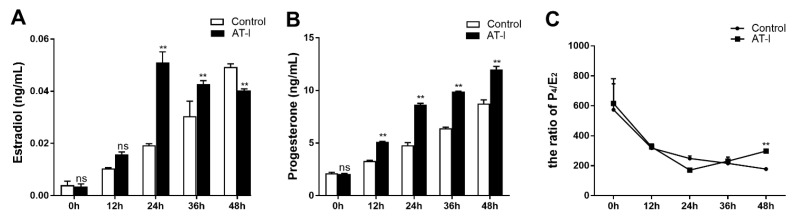
The effect of 10 μmol/L AT-I on the synthesis of E_2_ and P_4_ in FOGCs. (**A**) AT-I could significantly promote the E_2_ secretion in FOGCs at 24 h and 36 h, while the increased tendency weakened at 48 h. (**B**) The P_4_ concentration was significantly increased by AT-I treatment from 12 h to 48 h. (**C**) The ratio of P_4_/E_2_ indicated that the E_2_ secretion was more obviously increased than P_4_ secretion in FOGCs within 36 h after AT-I treatment. However, the secretion pattern of E_2_ and P_4_ changed at 48 h, in which the FOGCs secreted P_4_ more obviously than they secreted E_2_. Values are expressed as mean ± S.D. (n = 3). **, *p* < 0.01; ns, not statistically significant.

An amount of 10 µmol/L AT-I could significantly promote the E_2_ secretion in FOGCs at 24 h and 36 h, while the increased tendency weakened at 48 h (Figure 4A). The P_4_ concentration was significantly increased by AT-I treatment from 12 h to 48 h (Figure 4B). Although the secretion of both E_2_ and P_4_ was increased, the ratio of P_4_/E_2_ indicated that the E_2_ secretion was more obviously increased than P_4_ secretion in FOGCs within 36 h after AT-I treatment. However, the secretion pattern of E_2_ and P_4_ changed at 48 h, in which the FOGCs secreted P_4_ more obviously than they secreted E_2_ (Figure 4C).

### 3.5. Classification of Transcriptional Sequencing Data

To evaluate the transcriptional response of the FOGCs to AT-I exposure and to decipher the various host factors that may be involved in the luteinization, the Illumina HiSeq 4000 platform with cDNA libraries of FOGCs treated with AT-I was used. The sequencing quality data are shown in Table 1: the two libraries produced 58,841,865 and 52,950,059 original sequences (Raw Data), respectively, from both the treated and control FOGC groups. After filtering, the effective data of 58,189,124 and 52,340,848 were obtained. The proportion of data quality Q20 (sequencing base mass) of the two databases is more than 98%, which meets the needs of follow-up test analysis. All valid reads were aligned to the feline genome using Hisat 2.0. Additionally, from the treated and control groups of FOGCs, we obtained 56,730,426 and 51,014,192 mapped reads, respectively. Among these, the number of reads with unique alignment positions in the genome that met the requirements for subsequent experimental analysis were 53,563,083 and 48,058,826, respectively. The alignment ratios to the genome sequence were 92.06% and 91.82%, respectively. The outcomes demonstrate that the sequencing data possess a high level of quality and fulfill the criteria for additional analysis. (Table 2)

The sequencing data have been saved in the NCBI gene expression comprehensive database (http://www.ncbi.nlm.nih.gov/geo/info/linking.html, accessed on 1 July 2022.) and can be obtained by GEO Series accession number GSE155784.

### 3.6. Analysis of Differentially Expressed Genes

**Figure 5:**
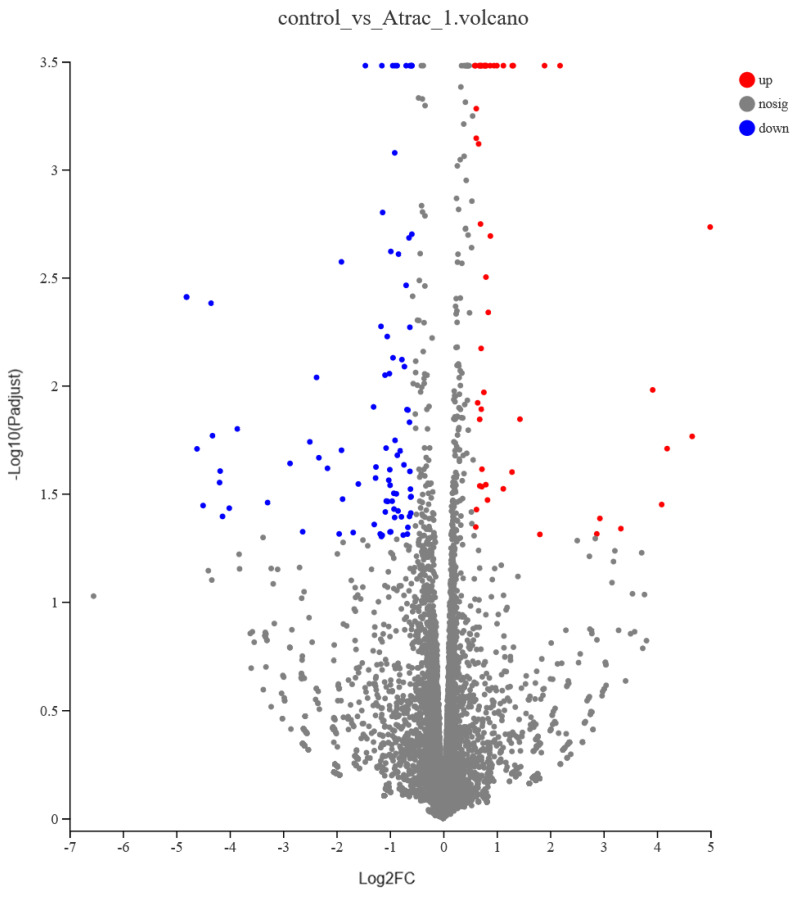
Volcano graph of 137 differentially expressed genes between two groups. Red represents up-regulated DEGs; blue represents down-regulated DEGs.

FOGCs treated with the AT-I showed a relative degree of differential expression. A total of 137 DEGs were obtained (≥1.5 fold change, *p-adjust* < 0.05), of which 49 DEGs were significantly up-regulated and 88 DEGs were significantly down-regulated. The overall distribution of DEGs can be understood by drawing a volcanic map (Figure 5).

### 3.7. Functional Enrichment Analysis of Differentially Expressed Genes

**Figure 6:**
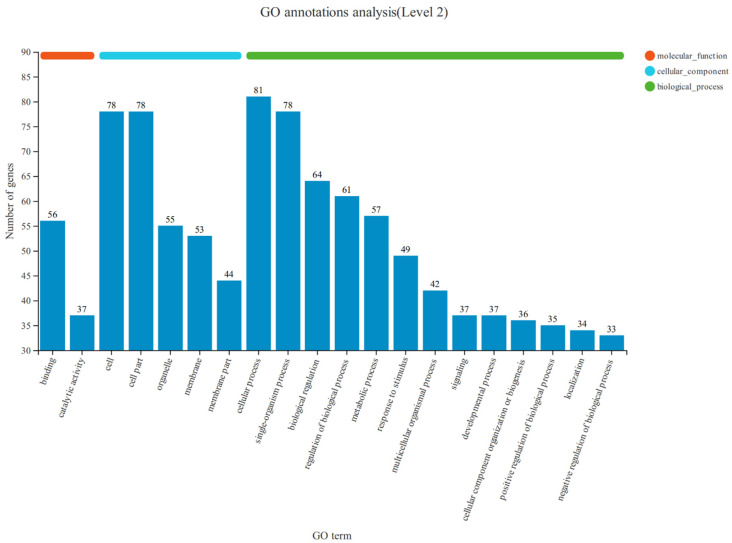
GO classifications of genes in FOGCs. The abscissa is the GO classification, and the ordinate is the number of genes in each category.

The RNA-seq analysis revealed a total of 17,265 genes, of which 137 genes showed significant differences in expression. To investigate the biological activities of the 137 DEGs, we performed a systematic enrichment analysis on Gene Ontology (GO) classification (accessible at http://www.geneontology.org/, accessed on 28 July 2022.) and the KEGG pathway (available at http://www.genome.jp/kegg/, accessed on 30 July 2022.). The annotated results of the GO database in the sequencing are shown in Figure 6. The histogram of GO enrichment analysis is mainly reflected in biological processes, cellular components, and molecular functions. In biological processes, 644 DEGs are assigned to the regulation of biological processes, such as cellular processes, single-organism process, biological regulation, regulation of biological process development, and metabolic processes. In the cell component field, 308 DEGs belong to the cell, the cell part, and membrane. In the molecular functional class, 93 DEGs resins can be used for binding and catalytic activity.

**Figure 7:**
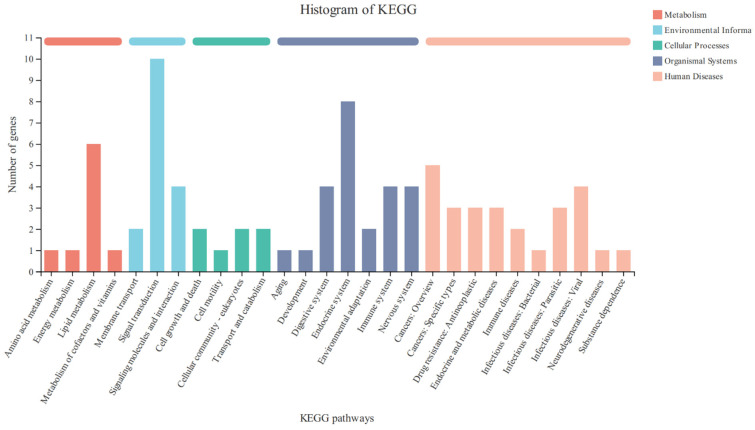
KEGG pathway classification of genes in FOGCs. The abscissa is the name of the KEGG metabolic pathway, and the ordinate is the ratio of the number of genes annotated to the pathway and the number of genes in the annotated genes.

**Figure 8:**
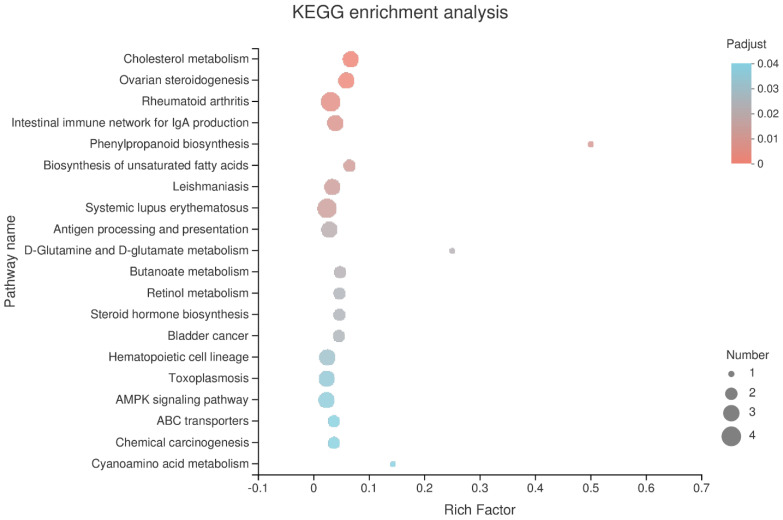
The significantly enriched KEGG pathway of DEGs. Rich factor, number of differentially expressed genes/total number of genes in this KEGG pathway. The larger the value, the greater the enrichment.

KEGG analysis showed that the DEGs were mainly involved in the regulation of the various signal transduction pathways, signaling molecules and their possible interactions, membrane transport, lipid metabolism, endocrine system, as well as the digestive system (Figure 7). The DEGs successfully annotated 155 signal pathways, and 23 signal pathways were significantly different (*p* < 0.05). Relatively high numbers of different genes were involved in the regulation of cholesterol metabolism, ovarian steroidogenesis, rheumatoid arthritis, biosynthesis of unsaturated fatty acids, steroid hormone biosynthesis, AMPK signaling pathway, ABC transporters, and other signaling pathways (Figure 8).

The DEGs were analyzed using KEGG to predict and investigate the signaling pathways that are mainly involved and most interesting, and to obtain those pathways that may be related to the luteinizing effect of AT-I. In the various pathways associated with the response of the FOGCs treated with AT-I, nine DEGs were found to be significantly affected (Table 3). Among the DEGs, three genes (*ABCA1*, *LDLR*, and *SREBF1*) were found to be up-regulated and involved in the regulation of the cholesterol metabolism. Cholesterol metabolism is a complex biological process and most of the other DEGs identified were also found to actively participate in this process. Three genes (*StAR*, *LDLR*, and *CYP1A1*) have been implicated in the regulation of ovarian steroidogenesis. Two genes (*SCD* and *FADS2*) were up-regulated and were found to be involved in the biosynthesis of unsaturated fatty acids. Two genes (*ABCG1* and *ABCA1*) were up-regulated and participated in ABC transporters, and the two DEGs (*SREBF1* and *SCD*) were observed to be up-regulated and could participate in the regulation of the AMPK signaling pathway.

### 3.8. Validation of the Selected Genes by Quantitative Reverse Transcription PCR and Biochemical Analysis

**Figure 9:**
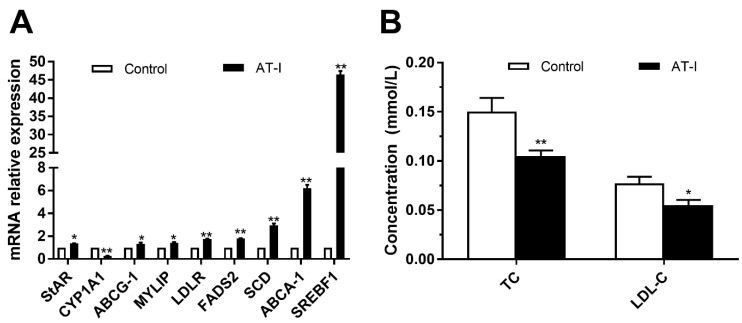
Verification of transcriptome results after 10 µmol/L AT-I treatment for 36 h. (**A**) Relative quantification of DEGs by RT-qPCR. The expression of each gene was normalized to the average expression of the endogenous reference gene GAPDH. (**B**) Biochemical verification. After AT-I treatment, the contents of total cholesterol and LDL-cholesterol both declined. The values are expressed as mean ± S.D. (n = 3). *, *p* < 0.05; **, *p* <0.01; ns, not statistically significant.

To validate the results of RNA-seq analysis, RT-qPCR was used to examine the various DEGs. All DEGs have the same trend of changes with RNA-seq data, thereby suggesting that the RNA-seq data reliably reflected the changes in the trends of gene expression (Figure 9A).

To verify whether AT-I can promote the metabolism of cholesterol, the biochemical analysis of the supernatants of granulosa cells treated with AT-I was carried out. It was found that the contents of the total cholesterol (TC) and low-density lipoprotein cholesterol (LDL-C) in the treated group with AT-I were significantly lower than those in the control group (Figure 9B).

## 4. Discussion

AT-I has been found to be the major bioactive component from AMK, which is a medicinal plant that has been used as a pharmacological agent for the treatment of threatened miscarriages in humans for a long time [4]. In most mammalian species the CL is responsible for P_4_ production during the early stages of pregnancy, but as pregnancy proceeds the placenta may take over steroid production. The identification of the P_4_ secretion source in cats varies among different studies. A study demonstrated that pregnant queens ovariectomized at day 30 or 45 of gestation exhibited a decline in serum P_4_ concentration and subsequently aborted; however, pregnancy was maintained by supplementation of the queens with synthetic progesterone after ovariectomy [22]. Braun BC et al. conclude that the feline placenta can produce P_4_ [14], suggesting that the feto-placental unit could be a source of gestational progesterone. So, it is currently widely believed that the CL is the main source of P_4_ in cats, but the placenta might locally support pregnancy via para- or autocrine mechanisms [13]. The CL is an essential organ for maintaining early pregnancy, and luteal dysfunction could cause infertility and abortions [9]. The CL is mainly composed of large luteum cells (LLCs) and a little amount of small luteum cells (SLCs). LLCs, which have a stronger steroidogenic capacity than SLCs, mainly originate from ovarian granulosa cells [23]. As the major bioactive component of AMK, the effect of AT-I on the ovarian granulosa cells might contribute to our understanding of AMK treatment of threatened miscarriages. This study found that AT-I could promote proliferation and the secretion of E_2_ and P_4_ in FOGCs. The transcriptome profile of differential gene expression in FOGCs treated with the AT-I was also examined in this report.

Ovarian granulosa cells are the largest cell group within the follicles. They not only cooperate with theca interstitial cells to regulate the synthesis of female steroid hormones, but also can regulate both the growth and maturation of oocytes [24]. At present, there is only one method to culture primary FOGCs, which is by slicing ovaries into many pieces [25], but the cell identification was not carried out and the fibroblasts and endothelial cells could not be effectively removed. In this study, a new method was used: puncturing small follicles to cultivate primary FOGCs. As confirmed with FSHR immunofluorescence identification, the FOGC purity could reach 90%, which could be used to perform relative experiments.

After ovulation, ovarian granulosa cells proliferated and transformed to LLCs, which can have the ability to secrete amounts of P_4_ and maintain early pregnancy [26,27]; Amelkina et al. revealed that ovarian granulosa cells could be transformed to LLCs, which can have the ability to secrete steroids in domestic cats [28]. In this study, the FOGC viability was noticed to increase significantly in a dose- and time-dependent manner within a certain range (Figure 3A,B) and the secretion of E_2_ and P_4_ increased significantly (Figure 4) after AT-I treatment in FOGCs. The increase in E_2_ and P_4_ might be due to cell proliferation or enhanced single-cell secretion or a combined effect, which needs further investigation.

Recent evidence obtained with a luteal cell line has confirmed that E_2_ can positively regulate the transcriptional activity of the *SR-BI* gene to speed up the occurrence of the luteinization process [29]. In our study, after AT-I-stimulation the E_2_ concentration was observed to increase in 36 h, and after AT-I treatment for 36 h FOGCs can produce a substantial amount of P_4_ instead of the E_2_ that they did before and the ratio of P_4_/E_2_ indicated that the P_4_ secretion was more obviously increased than E_2_ secretion in FOGCs after 36 h, indicating that the AT-I might initiate the luteinization process, which resulted in the enhanced P_4_ secretion in FOGCs.

In this study, a total of 137 DEGs were obtained after transcriptome sequencing on the FOGCs group treated with AT-I and these results were confirmed by RT-qPCR. The GO classification and KEGG pathway enrichment analysis have indicated that the DEGs are predominantly enriched in several significant pathways. These pathways are responsible for the regulation of cholesterol metabolism, ovarian steroidogenesis, the biosynthesis of unsaturated fatty acids, and the function of ABC transporters.

The unsaturated fatty acids are able to reduce the harmful cholesterol and triglyceride in the blood by esterifying cholesterol and promote cholesterol metabolism by effectively controlling the concentration of blood lipids, and increase the content of high-density lipoprotein (HDL), which is beneficial to the human body [30]. The DEG *SCD*, a central regulator controlling the biosynthesis of unsaturated fatty acids [31], was up-regulated by AT-I, which might contribute to the secretion of E_2_ and P_4_ through regulating the biosynthesis of unsaturated fatty acid.

The cholesterol metabolism is critical for the production of the various essential membrane components, which is necessary for cell proliferation [32]. In addition, as a precursor of steroid hormones, cholesterol is pivotal for ovarian follicular maturation [33]. Interestingly, in this study it was found that the DEGs after AT-I treatment mainly related to cholesterol metabolism. A constant supply of cholesterol is needed for the synthesis of steroid hormones in the CL and maternal cholesterol metabolism plays a role in fetal development [34]. Circulating plasma lipoproteins are the major source of cholesterol for steroid production in these different cells and cholesterol can be mainly obtained from circulating low-density lipoproteins (LDLs) and in small part from high-density lipoprotein (HDL) [32,35]. There are multiple systems involved in the cellular cholesterol delivery for steroidogenesis, mainly through the uptake of lipoprotein-derived cholesterol via LDLR-mediated endocytic pathways [36]. According to the RNA-seq analysis and RT-qPCR result, the expression of *LDLR* was induced by AT-I treatment in FOGCs, indicating that the uptake of lipoprotein-derived cholesterol might be activated, which could further stimulate the cholesterol biosynthesis. The identified DEG *SREBF1* that is responsible for encoding the sterol regulatory element-binding protein (SREBP) was able to promote the transcription of various lipogenesis involved in the biosynthesis of fatty acids and cholesterols and was involved in the regulation of sterol synthesis rates [27,34,37]. It was reported that SREBP could up-regulate the LDLR expression, promote the cholesterol uptake [33,38], and regulate the luteinization process through enhancing the sensitivity of human granulosa–lutein cells to LH [35]. After AT-I treatment, the *SREBF1* expression was up-regulated more than in the control group, suggesting that the SREBF1 signaling pathway might be activated. The activation of the SREBF1 signaling pathway by AT-I treatment might contribute to the FOGC synthesis of steroid hormones. To further confirm the effect of AT-I on cholesterol metabolism, the biochemical test was used to detect the content of cholesterol in the cell supernatant. After AT-I treatment for 36 h, the contents of total cholesterol and LDL cholesterol both declined, whereas the synthesis of steroid hormones increased, suggesting that AT-I indeed significantly affects cholesterol metabolism and promotes the secretion of E_2_ and P_4_ in FOGCs. Li et al. showed that the AT-I dose dependently inhibited Ox-LDL-induced VSMC proliferation to treat atherosclerosis [7], which might be the result of increased LDLR expression.

The ovaries are responsible for producing sex steroid hormones during reproductive life, which is important for both reproductive and somatic health [27]. After AT-I treatment, the secretion of E_2_ and P_4_ increased significantly in FOGCs. The *CYP1A1* was down-regulated and thereby can reduce the degradation of E_2_ to modulate ovarian steroidogenesis. The identified DEG *LDLR* is also a key gene in ovarian steroidogenesis by promoting the cholesterol biosynthesis. The DEG *StAR* can introduce cholesterol into mitochondria, which is essential for steroid production. Cholesterol is the major raw material for ovarian steroid hormone synthesis. According to this study, the increased expression of *LDLR*, *StAR*, and *SREBF1* was induced by AT-I treatment in FOGCs, and the increased gene expression might further stimulate the cholesterol biosynthesis by which leading ovarian steroidogenesis happened.

The increased *ABCA-1* and *ABCG-1* expression after AT-I treatment might improve the reverse cholesterol transport, and further speed up the metabolic process of cholesterol in FOGCs since the ABC transport system can drive intracellular superfluous cholesterol from arterial wall macrophages to the liver, thus allowing its excretion into the bile and feces as to speed up the metabolic process of cholesterol [39].

AT-I can promote the biosynthesis of unsaturated fatty acids to enhance the HDL content in plasma, and dramatically enhance the ability to transport LDL into cells by increased *LDLR* expression; the intake cholesterol can be used as raw material for ovarian steroidogenesis incloud E_2_ and P_4_. At the same time, AT-I can dynamically promote the reverse transport of cholesterol by up-regulating *ABCA1* and *ABCG-1* gene expression to speed up the metabolic process of cholesterol. Taken together, after AT-I treatment the differential genes identified were mainly concentrated on cellular cholesterol uptake and efflux. Thus, it was hypothesized that AT-I might affect the secretion of E_2_ and P_4_ by promoting the cholesterol metabolism in the granulosa cells.

## 5. Conclusions

This study demonstrated that AT-I could promote cholesterol metabolism determined by RNA-seq and biochemical testing in FOGCs. The effect of AT-I on cholesterol metabolism might help explain how AT-I induced the secretion of E_2_ and P_4_ in FOGCs. These results together will contribute to our understanding of the mechanism of early pregnancy protection by AMR.

## Figures and Tables

**Table 1:** Primers for RT-qPCR.

Gene Name	Nucleotide Sequence (5′-3′)	
GAPDH	Forward	CAAGGCTGTGGGCAAGGTCATC
	Reverse	TTCTCCAGGCGGCAGGTCAG
SREBF1	Forward	GGCATCGCAAGCAGGCTGAC
	Reverse	GGTGGGAGGTGGGCAGTGG
ABCG1	Forward	ACATGCTGTTGCCACACCTCAC
	Reverse	TCCTGCCTTCGTCCTTCTCCTG
ABCA1	Forward	GGCAACGGCACTGAGGAAGATG
	Reverse	TGCGGGAAAGAGGACTGGACTC
LDLR	Forward	GCCAGCAGAGGAGACGAGGAG
	Reverse	CCCGAAGCCCAGGAGGATGAG
SCD	Forward	AAATTCCCTTCGGCCAATGAC
	Reverse	TCTCACCTCCTCTTGCAGCAA
CYP1A1	Forward	TGGCACCATCAACAAGGCACTG
	Reverse	AAAGACCTCCAAGCGGGCAATG
FADS2	Forward	GGATATGCGGGCGTAGAAGC
	Reverse	GTGCCGTGCAAATAGGTGGA
StAR	Forward	GTGGAGCACATGGAAGCGATGG
	Reverse	GCAGCCAACTCGTGGGTGATG
MYLIP	Forward	AACGAGGGAGCAGGGTTGAA
	Reverse	ACACTGCCGAGACAGAGGTT

**Table 2:** Statistical summary analysis of RNA-seq data sets of treated cells and control cells.

Sample	Means for Raw Reads			Means for Mapping		
	No. of Raw Reads	No. of Valid Reads	Q20 (%)	No. of Map Reads	No. of Uniquely Mapped Reads	Uniquely Mapped Ratio (%)
Control cells	52,950,059	52,340,848	98.03	51,014,192	48,058,826	91.82
Treated cells	58,841,865	58,189,124	98.24	56,730,426	53,563,083	92.06

**Table 3:** Significantly up-regulated or down-regulated genes involved in signaling in FOGCs.

Gene Name	Gene Description	Fold Change	*p*-Adjust	Regulation
ABCA1	ATP binding cassette subfamily A member 1	4.554	0.000006	up
SCD	stearoyl-CoA desaturase	2.48	0.003099	up
LDLR	low-density lipoprotein receptor	1.934	0.025723	up
CYP1A1	cytochrome P450 1A1	0.451	0.026415	down
FADS2	fatty acid desaturase 2	1.609	0.034371	up
ABCG1	ATP binding cassette subfamily G member 1	3.724	0.044671	up
SREBF1	sterol regulatory element binding transcription factor 1	1.839	0.040339	up
StAR	steroidogenic acute regulatory protein	1.48	0.042111	up
MYLIP	myosin regulatory light chain interacting protein	2.451	0.002304	up

## Data Availability

All data are presented in this article, and the original data can be obtained by an email asking the author.

## Abbreviations

AMK	Atractylodes macrocephala Koidz
AT-I	atractylenolide I
FOGCs	primary feline ovarian granulosa cells
RNA-seq	transcriptome sequencing
CL	corpus luteum
LLCs	large luteum cells
SLCs	small luteum cells
E_2_	estradiol
P_4_	progesterone
ELISA	enzyme-linked immunosorbent assay
DEGs	differentially expressed genes
GO	Gene Ontology
KEGG	Kyoto Encyclopedia of Genes and Genomes
FC	fold change
FPKM	fragmented per kilobases of exon per million fragments mapped
RIN	RNA integrity number
RT-qPCR	quantitative reverse transcription PCR
DPBS	Dulbecco’s phosphate-buffered saline
DMEM/F12	Ham’s F-12 nutrient mixture
COCs	cumulus–oocyte complexes
HE	hematoxylin-eosin
FSHR	Follicle-stimulating hormone receptor
DAPI	4,6-diamidino-2-phenylindole
CCK-8	Cell Counting Kit-8
OD	value optical density value
TC	total cholesterol
LDL-C	low-density lipoprotein cholesterol
HDL-C	high-density lipoprotein cholesterol
SD	standard deviation
